# Elevated blood and cerebrospinal fluid biomarkers of microglial activation and blood‒brain barrier disruption in anti-NMDA receptor encephalitis

**DOI:** 10.1186/s12974-023-02841-7

**Published:** 2023-07-22

**Authors:** Haoxiao Chang, Jia Ma, Kai Feng, Ning Feng, Xinxin Wang, Jiali Sun, Tianshu Guo, Yuzhen Wei, Yun Xu, Huabing Wang, Linlin Yin, Xinghu Zhang

**Affiliations:** 1 Department of Neurology, Neuroinfection and Neuroimmunology Center, Beijing Tiantan Hospital, Capital Medical University, Beijing, 100070 China; 2 Department of Neurology, Beijing Shunyi Hospital, Beijing, 101300 China; 3 China National Clinical Research Center for Neurological Diseases, Beijing Tiantan Hospital, Capital Medical University, Beijing, 100070 China; 4 Department of Clinical Laboratory, Liaocheng Third People’s Hospital, Liaocheng, 252000 China; 5 Department of Critical Care Medicine, The First Affiliated Hospital of Henan University, Henan, 475001 China

**Keywords:** Anti-NMDA receptor encephalitis, Microglial activation, Blood‒brain barrier disruption, sTREM2, CD44, MMP9

## Abstract

**Background:**

Anti-NMDA receptor (NMDAR) encephalitis is an autoimmune disease characterized by complex neuropsychiatric syndrome and cerebrospinal fluid (CSF) NMDAR antibodies. Triggering receptor expressed on myeloid cells 2 (TREM2) has been reported to be associated with inflammation of the central nervous system (CNS). Matrix metalloproteinase-9 (MMP9) and cluster of differentiation (CD44) were measured to evaluate blood‒brain barrier (BBB) permeability in anti-NMDAR encephalitis. The roles of microglial activation and BBB disruption in anti-NMDAR encephalitis are not well known.

**Findings:**

In this work, we detected increased expression levels of CSF sTREM2, CSF and serum CD44, and serum MMP9 in anti-NMDAR encephalitis patients compared with controls. CSF sTREM2 levels were positively related to both CSF CD44 levels (*r* = 0.702, *p* < 0.0001) and serum MMP9 levels (*r* = 0.428, *p* = 0.021). In addition, CSF sTREM2 levels were related to clinical parameters (modified Rankin Scale scores, *r* = 0.422, *p* = 0.023, and Glasgow Coma Scale scores, *r* = − 0.401, *p* = 0.031).

**Conclusion:**

Increased sTREM2 levels in CSF as well as increased CD44 and MMP9 in serum and CSF reflected activation of microglia and disruption of the BBB in anti-NMDAR encephalitis, expanding the understanding of neuroinflammation in this disease. The factors mentioned above may have potential as novel targets for intervention or novel diagnostic biomarkers.

## Introduction

Anti-*N*-methyl-d-aspartate (NMDA) receptor (NMDAR) encephalitis is an autoimmune-mediated disease characterized by a complex neuropsychiatric syndrome, including rapidly progressive psychiatric symptoms or cognitive impairment, seizures, abnormal movements, or coma of unknown cause [1–3]. The disease is rare, with an annual incidence of 1.5 per million population, and has a female predominance (especially in individuals aged 12 to 45 years) [4, 5]. Both teenagers and adults with anti-NMDAR encephalitis often exhibit abnormal behavior with irritability and insomnia, followed by speech dysfunction, dyskinesias, memory deficits, autonomic instability, and decreased consciousness levels [3]. Seizures can occur at any phase during the disease and tend to occur earlier in males [6]. These psychiatric or behavioral symptoms make it difficult to distinguish anti-NMDAR encephalitis from a primary psychiatric disease. Most patients with anti-NMDAR encephalitis respond to immunotherapy. Second-line immunotherapy is usually effective when first-line treatments fail [5]. Progressive cerebellar atrophy is a prognostic marker of poor outcomes [7]. The only specific diagnostic biomarker of anti-NMDAR encephalitis is the presence of IgG antibodies against the GluN1 subunit of the receptor in the patient’s CSF [1]. Other diagnostic and prognostic biomarkers showed some potential utility. CXCL13 in cerebrospinal fluid (CSF) showed an increased concentration and was associated with a poor response to treatment in anti-NMDAR encephalitis patients [8]. Some inflammatory cytokines (TNF-α, IL-6, or IL-10) and YKL-40 (secreted protein expressed by microglia) showed an elevated level in the CSF of anti-NMDAR encephalitis patients, although the disease specificity of YKL-40 was unclear [9]. Soluble Fas and FasL in both CSF and serum showed increased levels in patients with anti-NMDAR encephalitis, but their prognostic implications are unclear [10]. Thus, the existing studies of biomarkers of anti-NMDAR encephalitis still have limitations, and more exploration is needed through retrospective or longitudinal clinical trials as well as studies of the pathogenesis of the condition. It is generally believed that antibodies against NMDARs are produced by tumors, viral infections, or other causes and act as inverse agonists of NMDARs on the surface of neurons in the central nervous system, leading to cross-linking and internalization of these receptors, which drives pathogenesis [1].

Recent brain biopsy and autopsy research and clinical evidence have shown that anti-NMDAR encephalitis patients at the acute stage (approximately 3 months or longer) have infiltration of immune cells, while neuroinflammation and the role of microglial activation have not yet been explored [11]. In addition, unlike other autoimmune-mediated demyelinating diseases, most patients at the recovery stage showed a large resolution of symptoms and a minimal presence of inflammation (both MRI changes or CSF pleocytosis) [1], which shows a lack of understanding of neuroinflammation in anti-NMDAR encephalitis.

The triggering receptor expressed on myeloid cells 2 (TREM2) is an immune receptor that is expressed abundantly by microglia in the central nervous system (CNS) and is involved in key immune-related functions of microglia activation [12–14]. Soluble TREM2 (sTREM2), released into the extracellular space (e.g., CSF and serum), is a soluble fragment of TREM2 detached by ADAM proteases [15]. Therefore, increased levels of sTREM2 represent an elevated inflammatory reaction [16].

Cluster of differentiation (CD) 44 is a type 1 transmembrane receptor, and its standard form is widely expressed in the majority of immune cells [17]. As a multifaceted receptor, CD44 exists in intracellular and soluble forms [18]. By mediating adhesion to extracellular matrix hyaluronan, CD44 recruits antigen-activated T lymphocytes and inflammatory agent-stimulated monocytes [19].

Matrix metalloprotease (MMP) 9, a Zn^2+^-dependent endopeptidase that can cleave type IV collagen in the extracellular matrix of the BBB, is induced by inflammatory cytokines, leading to disruption of the blood‒brain barrier (BBB) [20, 21]. Although disruption of the BBB is present in a variety of encephalitides, increased markers (CD44 and MMP9), which are reflected in BBB disruption in anti-NMDAR encephalitis, are first reported in our research.

In this study, we measured the concentrations of the above biomarkers in the CSF and serum of anti-NMDAR encephalitis patients to explore the relationship between microglial activation, BBB disruption, and anti-NMDAR encephalitis, providing insights into potential diagnostic markers and pathogenesis.

## Methods

### Study design, participants, and sample collection

In this study, 29 anti-NMDAR encephalitis and 15 control patients from the Neuroinfection and Neuroimmunology Center, Department of Neurology, Beijing Tiantan Hospital were enrolled between Jan. 2017 and Sep. 2021. The anti-NMDAR encephalitis patients were diagnosed according to the revised anti-NMDAR encephalitis diagnosis criteria of 2016 based on the clinical manifestations and identification of CSF antibodies against the GluN1 subunit of NMDAR by cell-based analysis [22]. The control patients (15 cases) with noninflammatory neurological diseases (OND) were selected, including benign intracranial hypertension (*n* = 7), peripheral neuropathy (*n* = 2), diabetic retinopathy (*n* = 1), intracranial hypotension headache (*n* = 1), anxiety disorders (*n* = 3), and hypertension (*n* = 1).

All samples were collected before treatment when anti-NMDAR encephalitis patients were in an acute phase. The CSF and serum samples were immediately centrifuged at 1000×*g* for 10 min and stored in the supernatant at − 80 °C until detection by ELISA**.**

### Clinical parameters

The clinical parameters in this study included demographic information (age, sex), adapted modified Rankin Scale (mRS), Glasgow Coma Scale (GCS), and Qalb. The patients’ evolution or functional outcome was evaluated using the GCS and mRS, and data were obtained from digital medical records.

The adapted modified Rankin Scale is an ordinal 7-point scale ranging from no symptoms (0) to severely disabled (5) or death (6) (Table 1). The mRS measures mobility (e.g., walking up and down a flight of stairs) and disability in basic (e.g., bathing, dressing) and instrumental (e.g., housekeeping, shopping) activities of daily living [23]. In this study, the mRS was conducted through a face-to-face structured interview. The mRS scores were used to assess the neurological status of each patient.

**Table 1:** Clinical and demographic characteristics of participants

Subject details	Con (*n* = 15)	Anti-NMDAR encephalitis (*n* = 29)
Age, mean ± SD, y^a^	43.13 ± 9.91	33.83 ± 13.87**
Sex, no. (%)^c^		
Male	4 (26.7%)	15 (51.72%)^ns^
Female	11 (73.3%)	14 (48.28%)
Adapted modified Rankin Scale (mRS) score		
Score 0, no symptoms	–	6 (20.69%)
Score 1, nondisabling symptoms	–	10 (34.49%)
Score 2, minor symptoms	–	1 (3.45%)
Score 3, moderate symptoms	–	3 (10.34%)
Score 4, moderately severe symptoms	–	3 (10.34%)
Score 5, severely disabled	–	6 (20.69%)
Score 6, dead	–	0
Glasgow Coma Scale (GCS)		
GCS 13–15, mild head injury	–	19 (65.50%)
GCS 9–12, moderate head injury	–	5 (17.25%)
GCS 3–8, severe head injury	–	5 (17.25%)
CSF WBCs (/μL), median (IQR)^a^	2.00 (1.00–3.00)	11.00 (4.50–21.50)***
CSF total protein (mg/dL), mean ± SD^b^	28.04 ± 1.83	34.47 ± 2.57*
CSF sTREM2 (pg/mL), median (IQR)^a^	24.75 (14.45–32.88)	49.15 (37.48–97.54)***
Serum sTREM2 (pg/mL), median (IQR)^a^	24.55 (13.32–34.73)	25.64 (17.50–42.92)^ns^
CSF CD44 (pg/mL), median (IQR)^a^	15.95 (12.09–72.15)	42.55 (31.33–69.47)*
Serum CD44 (pg/mL), median (IQR)^b^	7.72 (4.26–12.17)	13.2 (10.92–17.33)***
Serum MMP9 (pg/mL), median (IQR)^a^	210.63 (179.56–268.60)	306.09 (209.68–527.40)*
Qalb (mean ± SD, damage %)	3.89 ± 2.66	5.64 ± 2.92
MRI contrast enhancement (%)	1 (6.67%)	20 (68.97%)

Data are expressed as the mean ± SD or median ± SD (IQR) for continuous variables

*y* year, *CSF* cerebrospinal fluid, *WBCs* white blood cells, *sTREM2* soluble triggering receptor expressed on myeloid cells-2, *CsD44* cluster of differentiation 44, *MMP9* matrix metalloproteinase-9, *Qalb* cerebrospinal fluid (CSF)/serum albumin quotient

^a^Assessed by the Mann‒Whitney test

^b^Assessed by the independent two-sample *t* test

^c^Assessed by Chi-squared analysis with Yates’ continuity correction. *p < 0.05, ***p < 0.001, ns,* p* > 0.05

The Glasgow Coma Scale is an essential and well-validated coma scale in critical care for rapidly determining the neurological status of patients and for estimating their long-term prognosis [24]. The scale included three major responses, and each level of response was assigned a number: the worse the response, the lower the number: eye opening (E), verbal response (V), and best motor response (M). The GCS was applied to subdivide the severity: a score of 8 was used to signify a severe head injury; scores of 9–12 were designated as moderate injury; and scores of 13–15 were classified as mild head injury [25].

The cerebrospinal fluid (CSF)/plasma albumin ratio (Qalb) is believed to reflect the integrity of the blood‒brain barrier (BBB) [26]. As Qalb is age dependent, the individual age-related reference value was defined as Qalb* = (4 + Age/15) × 10^−3^. We calculated the Qalb (Alb_CSF_/Alb_serum_) in all patients, and an elevated Qalb (Qalb > Qalb*) was defined as disruption of the BBB [27].

### Ethics approval and consent to participate

Experiments were carried out following the ethical principles established in the Declaration of Helsinki. Patients (or their representatives) were informed about this study and gave written informed consent. The study was approved by the Ethics Committee of Beijing Tiantan Hospital, Capital Medical University (No. KY2015-031-02). All participants provided written informed consent before proceeding with the study.

### Detection of biomarkers by ELISA

Enzyme-linked immunosorbent assay (ELISA) kits were applied to measure the concentration of sTREM2 (Cat. ab224881, Abcam), CD44 (Cat. CSB-E11846h, Cusabio), and MMP9 (Cat. CSB-E08006h, Cusabio) in humoral fluids. All detections and measurements were performed following the manufacturer’s instructions, and every standard and sample was assayed in duplicate.

### Statistical analysis

All statistical analyses were conducted using SPSS version 24.0 (IBM, Armonk, NY, US) and Prism (GraphPad software 8.0 version). Data are presented as the mean ± SD or the median with an interquartile range based on the normality test. The differences in CSF total protein concentrations and serum CD44 levels between the anti-NMADR encephalitis and control groups were assessed with an independent two-sample *t* test. The Mann‒Whitney *U* test was applied when comparing the differences in age, CSF white blood cell (WBC) counts, CSF CD44 levels, CSF sTREM2, and serum MMP9 levels between the anti-NMDAR encephalitis and control groups. Differences in both CSF sTREM2 and CSF CD44 levels between the good clinical outcome group and the poor clinical outcome group in anti-NMDAR encephalitis were assessed by the Mann‒Whitney *U* test. Statistical analysis of CSF sTREM2, serum sTREM2, serum MMP9, serum CD44, and CSF CD44 levels was performed after adjusting the confounding factors, age, and sex in the multivariate linear regression analyses. The differences in sex between the control group and anti-NMDAR encephalitis were performed with Chi-squared analysis with Yates’ continuity correction. Correlation coefficients between sTREM2, CD44, MMP9, mRS scale, GCS scores, and Qalb were calculated using Spearman’s two-tailed correlation test when one of the variables did not satisfy a normal distribution. A *p* value < 0.05 was considered statistically significant.

## Results

### Patient characteristics

The clinical data and the concentrations of the biomarkers from patients with anti-NMADR encephalitis (*n* = 29, 15 male and 14 female) and OND controls (*n* = 15, 4 male and 11 female) are presented in Table 1. Diagnoses of anti-NMDAR encephalitis were confirmed by two doctors according to the revised diagnostic criteria (2016). All OND controls were negative for specific CSF antibodies. Age in the anti-NMDAR encephalitis group was 33.83 ± 13.87 and 43.13 ± 9.91 in the control group, which showed a slight decrease in the anti-NMDAR encephalitis group (*p* = 0.006, Table 1) compared with the control group. There were no notable differences in gender proportion between the anti-NMDAR encephalitis group and the control group. The CSF WBC counts were 11.00 (4.50–21.50)/μL in the anti-NMDAR encephalitis group and 2.00 (1.00–3.00)/μL in the control group. The CSF WBC counts showed an increase in the anti-NMDAR encephalitis group compared with the control group (*p* < 0.001, Table 1). The CSF total protein concentration was 34.47 ± 2.57 in the anti-NMDAR encephalitis group and 28.04 ± 1.83 in the control group. The CSF total protein showed a higher concentration in the anti-NMDAR encephalitis group (*p* = 0.048, Table 1) than in the control group. The increase in the CSF WBC count and CSF total protein concentration in anti-NMDAR encephalitis patients hints at neuroinflammation. The mRS scale was used to measure the degree of disability or dependence in daily activities, and the GCS score was applied to assess consciousness level. The Qalb was used to measure the degree of BBB permeability. Brain MRI findings showed that 20 patients had contrast enhancement in the anti-NMDAR encephalitis group and one patient in the control group (Table 1).

### sTREM2, MMP9, and CD44 are increased in the extracellular fluid of anti-NMDAR encephalitis patients

**Fig. 1:**
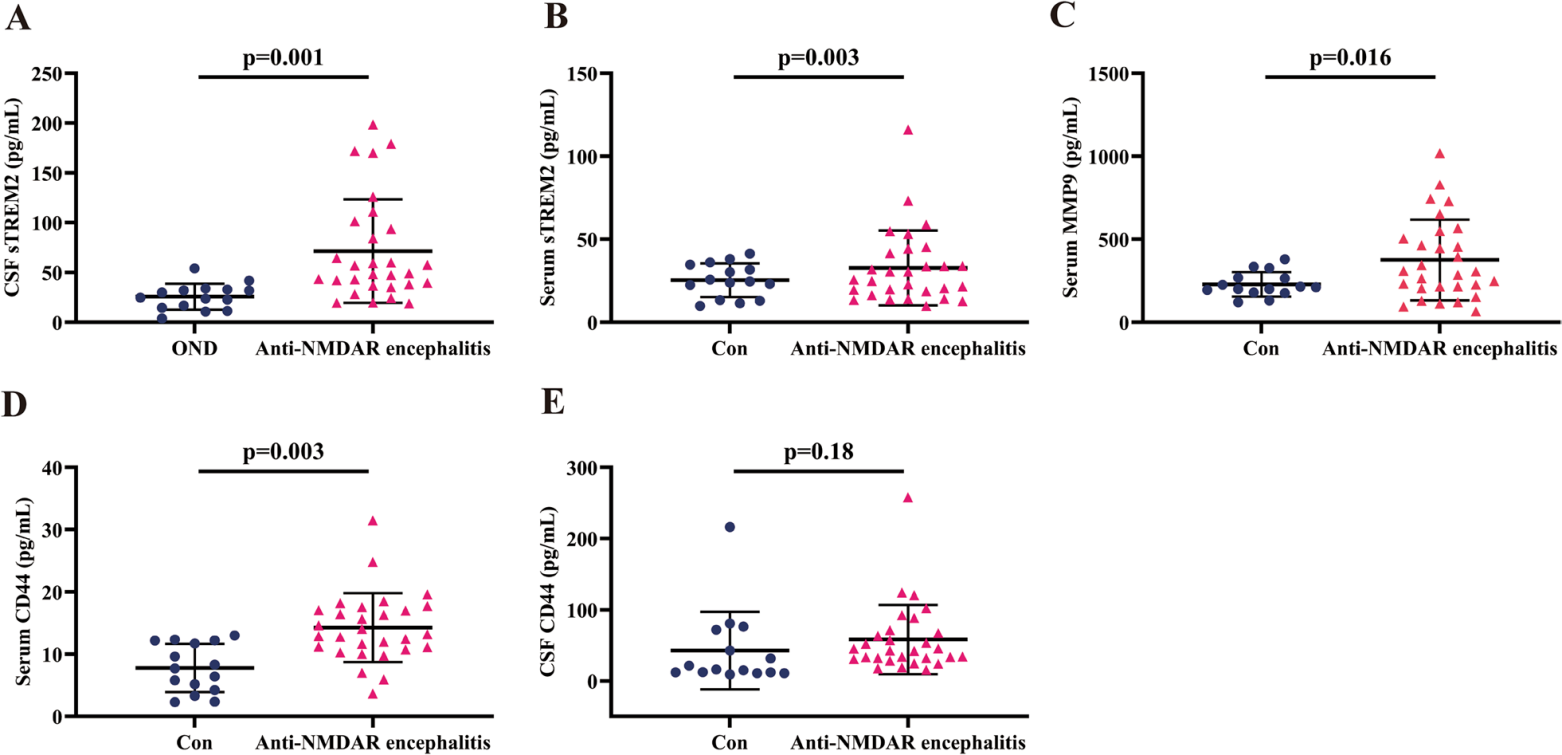
Comparison of sTREM2, MMP9, and CD44 levels in serum and CSF between anti-NMDAR encephalitis patients and controls. **A**, **B** The CSF sTREM2 levels were significantly increased (*p* < 0.001) in the anti-NMDAR encephalitis group, and the serum sTREM2 levels also showed an increasing trend (*p* = 0.003) compared with the controls. **C** The serum MMP9 levels were higher (*p* = 0.016) in the anti-NMDAR encephalitis group than in the controls. **D**, **E** CD44 levels in both the serum (**D**, *p* = 0.003) and the CSF (**E**, *p* = 0.18) were significantly higher in the anti-NMDAR encephalitis group than in the control group. Differences in CSF sTREM2 levels, serum sTREM2 levels, serum MMP9 levels, and CSF CD44 levels were assessed by the Mann‒Whitney test between the anti-NMDAR encephalitis group and the control group. Differences in serum CD44 levels were assessed by the independent two-sample *t* test. The above statistical results have been adjusted for age and sex

The median levels of CSF sTREM2 were 49.15 (37.48–97.54) ng/mL in the anti-NMDAR encephalitis group and 24.75 (14.45–32.88) ng/mL in the OND control group. The median levels of serum sTREM2 were 25.64 (17.50–42.92) ng/mL in the anti-NMDAR encephalitis group and 24.55 (13.32–34.73) ng/mL in the control group. The level of CSF sTREM2 was increased significantly in the anti-NMDAR encephalitis patients compared with the control group (Fig. 1A, *p* = 0.001), and the serum sTREM2 levels showed an increase in the anti-NMDAR encephalitis patients (Fig. 1B, *p* = 0.003).

The median levels of serum MMP9 were 306.09 (209.68–527.40) ng/mL in the anti-NMDAR encephalitis group and 210.63 (179.56–268.60) ng/mL in the control group. The serum MMP9 levels increased markedly in the anti-NMDAR encephalitis group compared with the control group (Fig. 1C, *p* = 0.016).

In addition, the median levels of CSF CD44 were 42.55 (31.33–69.47) ng/mL in the anti-NMDAR encephalitis group and 15.95 (12.09–72.15) ng/mL in the control group. The median levels of serum CD44 were 13.2 (10.92–17.33) ng/mL in the anti-NMDAR encephalitis group and 7.72 (4.26–12.17) ng/mL in the control group. Significantly elevated CD44 levels in CSF (Fig. 1D, *p* = 0.003) were observed between the anti-NMDR encephalitis group and the control group, while the CD44 levels in serum showed no marked differences (Fig. 1E, *p* = 0.18).

### sTREM2 levels in anti-NMDAR encephalitis patients correlated with inflammatory factors and clinical parameters

**Fig. 2:**
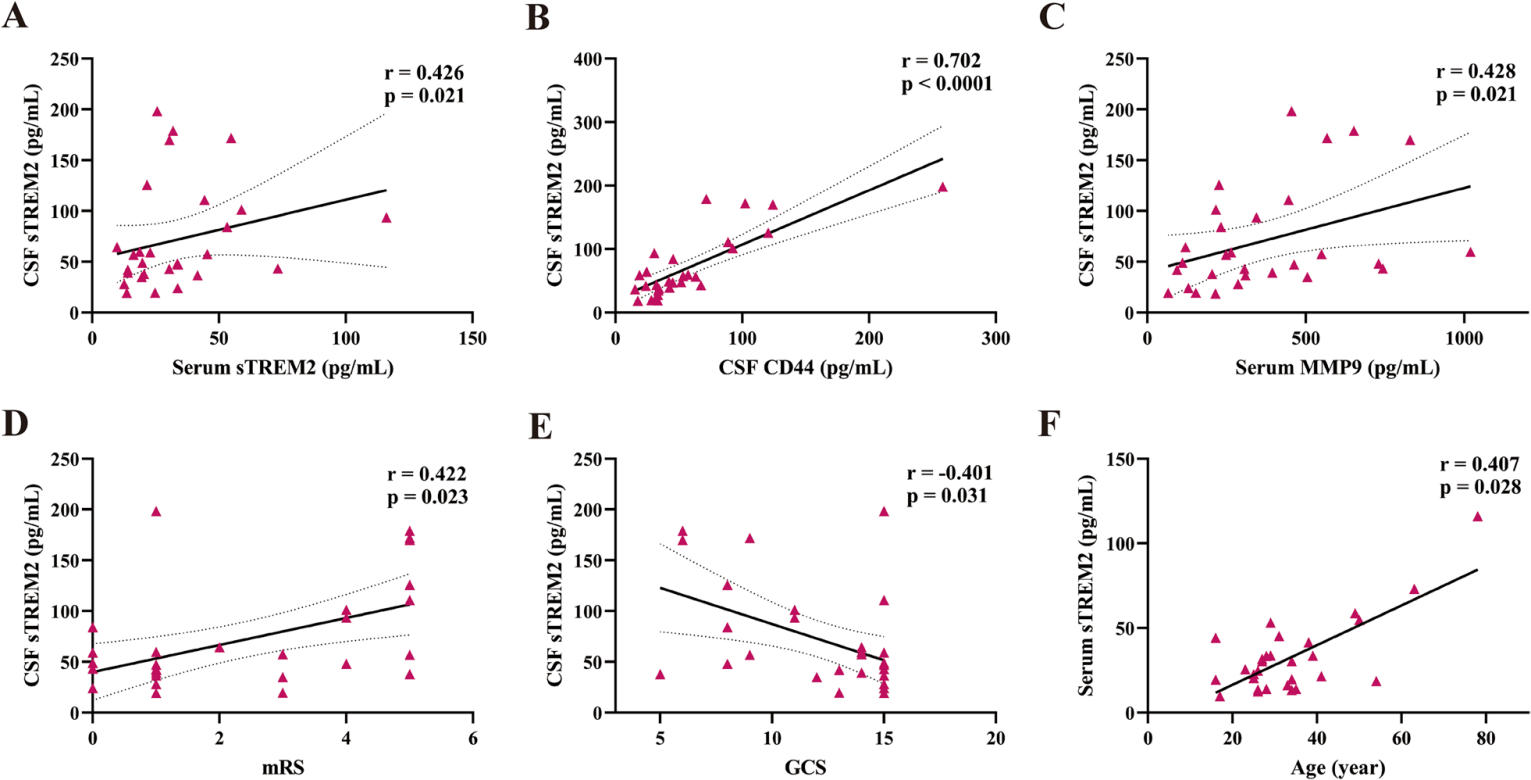
Correlation analysis of sTRME2 levels in CSF or serum with other parameters. **A**–**C** The CSF sTREM2 level was positively related to the serum sTREM2 level (*r* = 0.426, *p* = 0.021), the CSF CD44 level (*r* = 0.702, *p* < 0.0001), and the serum MMP9 level (*r* = 0.428, *p* = 0.021). **D**–**F** The CSF sTRME2 level was positively related to the mRS scale (*r* = 0.422, *p* = 0.023) and age (*r* = 0.407, *p* = 0.028) but negatively related to the GCS score (*r* = − 0.401, *p* = 0.031). Spearman correlation coefficients were used for the analysis

CSF sTREM2 is related to anti-NMDAR encephalitis inflammatory factors. As shown in Fig. 2A, a significant positive correlation was found between the CSF sTREM2 expression level and the serum sTREM2 level (*r* = 0.426, *p* = 0.021, Fig. 2A). A similar positive correlation was shown between the CSF sTREM2 expression level and the CSF CD44 (*r* = 0.702, *p* < 0.0001, Fig. 2B) and between the CSF sTREM2 expression level and the serum MMP9 expression levels (*r* = 0.428, *p* = 0.021, Fig. 2C) in the anti-NMDAR encephalitis patients. In addition, the CSF and serum sTREM2 expression levels are related to the clinical parameters. Regarding the CSF sTREM2 expression level, there was a significant positive correlation between the CSF sTREM2 expression level and mRS scale (*r* = 0.422, *p* = 0.023, Fig. 2D) and a negative correlation between the CSF sTREM2 expression level and GCS scale (*r* = − 0.401, *p* = 0.031, Fig. 2E) in the anti-NMDAR encephalitis patients. Regarding the serum sTREM2 expression level, the serum sTREM2 expression level was notably positively correlated with age in anti-NMDAR encephalitis patients (*r* = 0.407, *p* = 0.028).

### Both CSF CD44 and serum MMP9 are correlated with clinical parameters

**Fig. 3:**
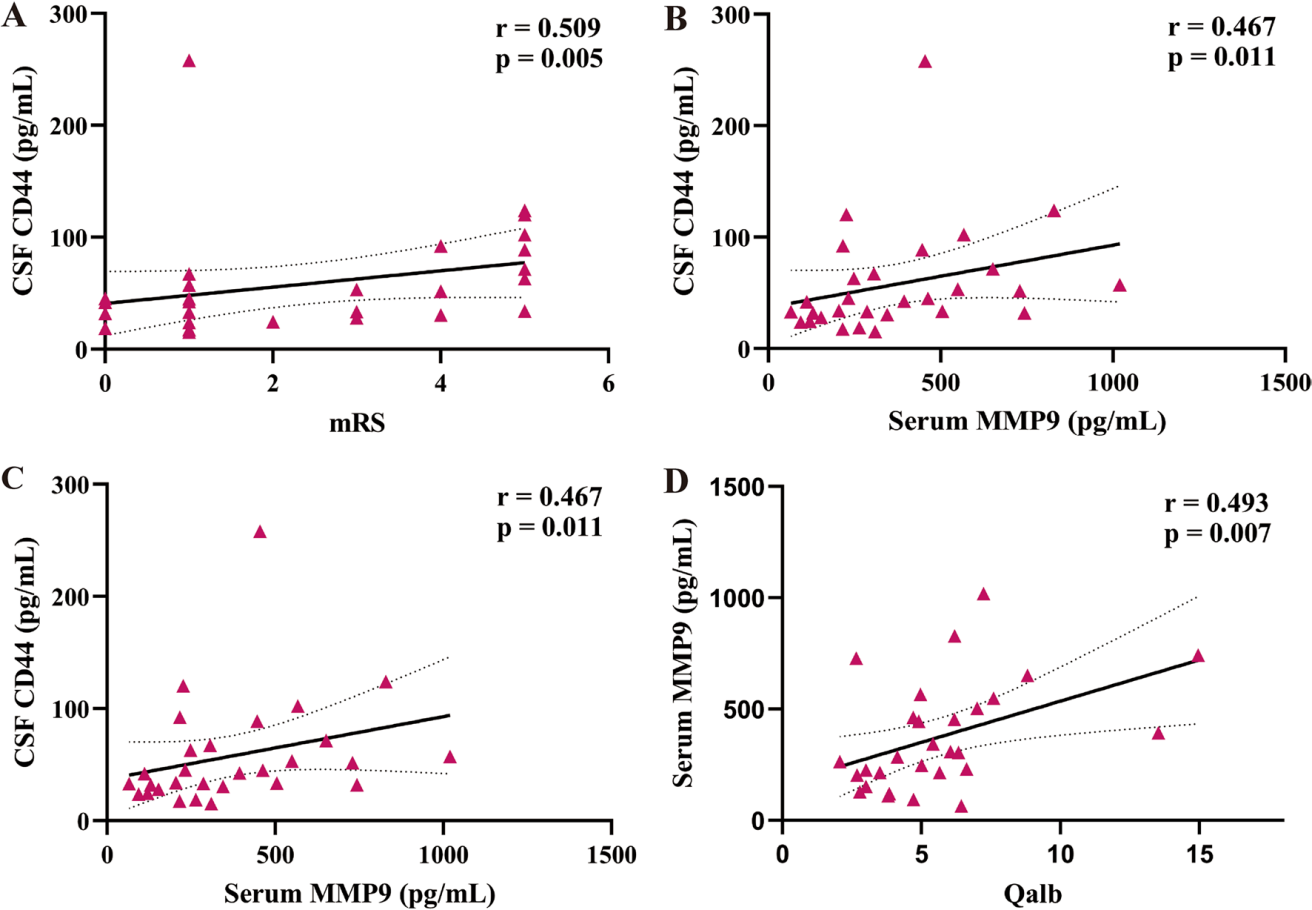
Correlation analysis of CSF CD44 levels with clinical parameters and serum MMP9 levels. **A**, **B** The CSF CD44 level was positively related to the mRS scale (*r* = 0.509, *p* = 0.005) and negatively related to the GCS score (*r* = − 0.382, *p* = 0.041). **C** The CSF CD44 level was positively related to the serum MMP9 level (*r* = 0.467, *p* = 0.011). **D** The serum MMP9 level is positively related to the Qalb value (*r* = 0.493, *p* = 0.007). Spearman correlation coefficients were used for the analysis

Other inflammatory factors (CD44 and MMP9) are related to clinical parameters, including mRS, GCS, and Qalb. For the CSF CD44 expression level, a positive correlation was shown between the CSF CD44 expression level and the mRS scale (*r* = 0.509, *p* = 0.005, Fig. 3A), and a negative correlation was found between the CSF CD44 expression level and GCS scale (*r* = − 0.382, *p* = 0.041, Fig. 3B). There was also a positive correlation between the CSF CD44 expression level and the serum MMP9 expression level (*r* = 0.467, *p* = 0.011, Fig. 3C). For the serum MMP9 expression level, a positive correlation was demonstrated between the serum MMP9 expression level and the Qalb in anti-NMADR encephalitis patients (*r* = 0.493, *p* = 0.007, Fig. 3D).

### CSF sTREM2 shows a better predictive value in clinical outcomes

**Fig. 4:**
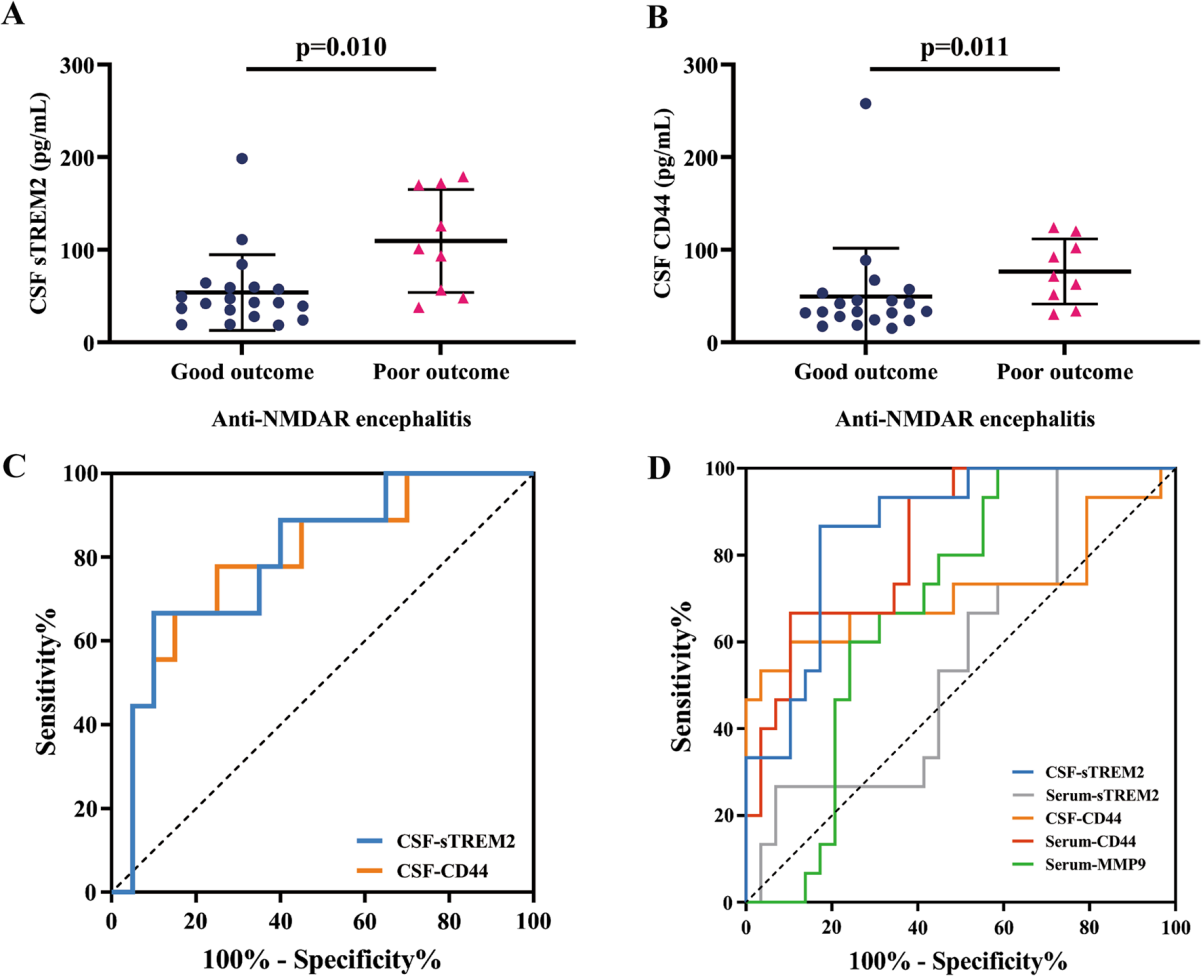
The predictive value of CSF sTREM2 and CSF CD44 for anti-NMDAR encephalitis. **A**, **B** Each patient’s clinical outcome was classified as good or poor according to the mRS score (mRS > 3 poor outcome, mRS ≤ 3 good outcome). In anti-NMDAR encephalitis, both CSF sTREM2 (*p* = 0.010) and CSF CD44 (*p* = 0.011) showed higher concentrations in patients with poor outcomes than in patients with good outcomes. **C** Receiver operating characteristic curve (ROC) analysis for CSF sTREM2 (AUC = 0.800, *p* = 0.009) and CSF CD44 (AUC = 0.794, *p* = 0.0125) in distinguishing good and poor clinical outcomes in anti-NMDAR encephalitis. **D** Receiver operating characteristic curve (ROC) analysis was applied to determine the diagnostic value of sTREM2 (CSF sTREM2 AUC = 0.864, *p* < 0.0001; serum sTREM2 AUC = 0.568, *p* = 0.4651), CD44 (CSF CD44 AUC = 0.729, *p* < 0.018; serum CD44 AUC = 0.837, *p* = 0.0003), and MMP9 (serum MM9 AUC = 0.687,* p* = 0.0436) in anti-NMDAR encephalitis. The Mann‒Whitney *U* test was applied when comparing the differences in CSF sTREM2 and CSF CD44 between the good outcome group and poor outcome group in anti-NMDAR encephalitis

We defined the clinical outcome according to mRS scores (3, moderate disability, as the diving point; > 3 poor outcome, ≤ 3 good outcome). In the anti-NMDAR encephalitis patients, the CSF sTREM2 levels in patients with good outcomes were lower than those in patients with poor outcomes (*p* = 0.011, Fig. 4A). The levels of CSF CD44 were also slightly decreased in the patients with good outcomes (*p* = 0.011, Fig. 4B) compared with the patients with poor outcomes in anti-NMDAR encephalitis. The levels of serum sTREM2 (*p* = 0.153), serum CD44 (*p* = 0.940), and serum MMP9 (*p* = 0.295) showed no differences in patients with a good outcome compared with patients with a poor outcome in anti-NMDAR encephalitis. These results showed that CSF sTREM2 levels and CSF CD44 levels had different distributions in the prognosis of anti-NMDAR encephalitis.

To further evaluate the ability of CSF sTREM2 levels and CSF CD44 levels to indicate the severity of neurologic impairments in anti-NMADR encephalitis patients, we performed receiver operating characteristic curve (ROC) analysis. We found that both CSF sTREM2 levels and CSF CD44 levels showed predictive value in the clinical outcome of anti-NMDAR encephalitis. The area under the curve (AUC) for CSF sTREM2 in anti-NMDAR encephalitis patients to distinguish from poor clinical outcome was 0.800 (*p* = 0.0109), and the AUC for CSF CD44 was 0.794 (*p* = 0.0125, Fig. 4C). These results confirmed that CSF sTREM2 levels had a better predictive value in distinguishing the clinical outcome in anti-NMDAR encephalitis patients.

In addition, we also applied ROC analyses to show the diagnostic value of sTREM2, CD44, and MMP9 in CSF or serum for anti-NMDAR encephalitis (Fig. 4D). The CSF sTREM2 levels could differentiate anti-NMDAR patients (*N* = 29) well from the control group (*N* = 15), with an AUC of CSF sTREM2 levels of 0.864 (*p* < 0.0001). The AUC for CSF CD44 levels was 0.729 (*p* = 0.018), for serum CD44 levels was 0.837 (*p* = 0.0003), and for serum MMP9 levels was 0.687 (*p* = 0.0436), while the serum sTREM2 level showed a poor diagnostic value with an AUC of 0.568 (*p* = 0.4651).

## Discussion

This study aims to show that activated microglia in anti-NMDAR encephalitis are accompanied by BBB disruption and are closely related to clinical features. The results indicated that (1) the increased concentrations of sTREM2, CD44, and MMP9 in the CSF and serum of the anti-NMDAR encephalitis group compared with the control groups; (2) the above biomarkers are related to clinical parameters (mRS scale, GCS score, and Qalb); (3) CSF sTREM2 and CSF CD44 have predictive value in distinguishing the severity of clinical outcome; and (4) the activation of microglia and disruption of the BBB play a role in the pathogenesis of anti-NMDAR encephalitis.

The soluble form of TREM2 (sTREM2) is reported to be increased in the cerebrospinal fluid of patients with Alzheimer’s disease, multiple sclerosis, and neurosyphilis as a marker of microglial activation during neuroinflammation [28–33]. Zhong et al. found that sTREM2 modulates microglial functions to reduce amyloid plaque load and rescues functional deficits in spatial memory and long-term potentiation in Alzheimer’s disease (AD) [34]. In cohort studies of Alzheimer’s disease, the concentration of CSF sTREM2 in patient samples increased with an association with tau pathology and neurodegeneration, especially in the early stage [35, 36]. Thus, due to the dynamic inflammatory changes in AD, the detection of sTREM2 in CSF and serum has become a potential tool to track the progression of AD in clinical management [37]. In inflammatory demyelinating diseases, such as multiple sclerosis (MS), increased CSF sTREM2 levels are associated with neuroinflammation [38]*.* However, very few studies have detected sTREM2 levels in the CSF and serum of patients with anti-NMDAR encephalitis. Based on the correlation between sTREM2 and inflammation, our study aims to explore the possibility of sTREM2 as a biomarker of anti-NMADR encephalitis, and our results also confirm the hypothesis.

Activation of microglia promotes the production of cytokines, chemokines, and matrix metalloproteinases [14], which disrupt the blood‒brain barrier and lead to the entry of blood-derived immune cells, cytokines, and supplements into the central nervous system, further activating microglia and increasing neuronal damage [39]. Tobias Zrzavy et al. confirmed a topographic distribution of inflammation in two untreated anti-NMDAR encephalitis patients, accompanied by infiltrated immune cells (CD3+/CD8+ T cells and CD79a+ B cells/plasma cells) [40]. However, brain MRI showed a lower frequency of abnormal MRI findings [1]. In this study, we found that the concentration of sTREM2 was increased in the CSF of anti-NMDAR encephalitis patients compared with controls, showing that microglial activation may play a role in pathogenesis in the acute stage of anti-NMDAR encephalitis.

Previous studies have shown that a damaged BBB is related to a poor prognosis in anti-NMDAR encephalitis [41]. To further explore this hypothesis, we found that CD44 and MMP9 levels were also increased in CSF and serum, respectively. The CSF sTREM2 level is positively correlated with both CSF CD44 levels and serum MMP9 levels. In the inflammatory environment, CD44 is upregulated and secreted by M1 macrophages and modulates leukocyte adhesion, migration, and functional phenotype [19]. In addition, CD44 is regulated by hyaluronan, a key biophysical component of the BBB, impairing the barrier integrity of brain microvascular endothelial cells through a CD44-dependent pathway [42]. Furthermore, studies have shown that MMP-9 can cause leakage of the BBB [20]. The level of serum MMP9 was related to Qalb, a common clinical indicator to evaluate the destruction of the BBB, in anti-NAMDR encephalitis patients. Thus, the high levels of CSF CD44 and serum MMP9 in anti-NMDAR encephalitis patients showed evidence of [18] BBB disruption in anti-NMDAR encephalitis, accompanied by microglial activation. Since disruption of the BBB is a poor prognostic factor of anti-NMDAR encephalitis, the measurement of CD44 and MMP9 in CSF and serum can be used as a potential biomarker to indicate its prognosis in the clinical arrangement. In our study, CSF CD44, serum CD44, and serum MMP9 levels had diagnostic value in distinguishing anti-NMDAR encephalitis (Fig. 4D). mRS scales and GCS scores are used to estimate the severity of clinical characteristics in anti-NMDAR encephalitis patients. The levels of both CSF sTREM2 and CD44 were positively related to the mRS scores and negatively related to the GCS scores, which showed the potential for indicating the severity of anti-NMDAR encephalitis. Furthermore, the CSF sTREM2 and CSF CD44 levels can serve as predictive biomarkers to classify clinical outcomes (Fig. 4C).

Above all, from the perspective of neuroinflammation, our study emphasized the evidence of microglial activation and disruption in anti-NMDAR encephalitis. The results showed that microglia play a role in pathogenesis and that the damaged BBB is involved in pathology, which provides a new perspective on potential targets for treatment and biomarkers for diagnosis.

## Data Availability

The raw data supporting the conclusions of this article are available from the corresponding author upon reasonable request.

## Abbreviations

sTREM2: Soluble triggering receptor expressed on myeloid cells 2
CSF: Cerebrospinal fluid
MMP9: Metalloproteinase-9
CD44: Cluster of differentiation 44
BBB: Blood‒brain barrier
NMDAR: *N*-Methyl-d-aspartate receptor
